# A Dangerous Duo: Complete Heart Block Triggered by Anterior Wall Myocardial Infarction

**DOI:** 10.7759/cureus.102642

**Published:** 2026-01-30

**Authors:** Lea Moujaes, Jace Bradshaw, Ubah Dimbil, Rishab Agarwal, P. Logan Weygandt

**Affiliations:** 1 Emergency Medicine, Johns Hopkins University School of Medicine, Baltimore, USA; 2 Emergency Medicine/Anesthesiology and Critical Care, Johns Hopkins University School of Medicine, Baltimore, USA; 3 Emergency Medicine, Inova Fairfax Hospital, Falls Church, USA; 4 Cardiac Surgery, Johns Hopkins University School of Medicine, Baltimore, USA

**Keywords:** anterior stemi, cardiogenic shock, complete heart block, coronary revascularization, left anterior descending artery occlusion

## Abstract

Complete heart block (CHB) is an uncommon but life-threatening complication of anterior ST-elevation myocardial infarction (STEMI). We report a case of a 62-year-old male who presented to the emergency department with severe chest pain, profound bradycardia, and an undetectable blood pressure. Initial electrocardiogram demonstrated complete atrioventricular dissociation, and emergent transcutaneous pacing and vasopressor support were initiated. The patient subsequently developed ventricular tachycardia degenerating into recurrent conduction abnormalities with evolving ST-segment elevation. Emergent coronary angiography revealed a proximal left anterior descending (LAD) artery occlusion, and percutaneous coronary intervention with drug-eluting stent placement was performed. A transvenous pacemaker and an intra-aortic balloon pump were placed to stabilize cardiogenic shock. This case highlights the rarity and severity of CHB caused by proximal LAD occlusion, emphasizing the importance of rapid rhythm stabilization, early recognition of ischemic conduction disturbances, and immediate revascularization. Clinicians should maintain a high index of suspicion for conduction system compromise even in anterior infarction, as timely intervention is critical to preventing irreversible hemodynamic collapse.

## Introduction

Complete heart block (CHB) is a critical complication of acute myocardial infarction (AMI), typically arising from right coronary artery occlusion leading to inferior wall ischemia and subsequent atrioventricular (AV) node dysfunction [1]. In contrast, acute anterior wall myocardial infarction (AWMI) secondary to a proximal left anterior descending (LAD) artery occlusion is a relatively rarer etiology of CHB [1]. However, the latter is associated with significantly higher morbidity and mortality due to the extensive myocardial damage and resulting hemodynamic instability [1].

Conduction disturbances in AWMI typically involve the distal conduction system (bundle of His and bundle branches), making the presentation of CHB an immediate and severe clinical challenge. This report details the case of a patient who presented with profound cardiogenic shock secondary to CHB as the primary manifestation of a proximal LAD occlusion, underscoring the necessity of rapid recognition, aggressive hemodynamic support, and emergent revascularization in this high-risk patient cohort.

## Case presentation

**Figure 1:**
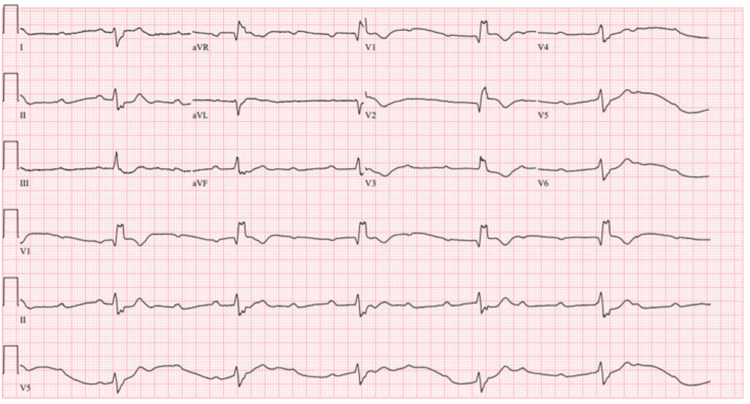
ECG demonstrating complete heart block. This ECG shows complete dissociation between atrial and ventricular activity. P waves are present and occur at a regular rate, representing ongoing atrial depolarization, but they are not consistently followed by QRS complexes because no atrial impulses are conducted through the atrioventricular (AV) node. The QRS complexes are also regular but occur at their own intrinsic rate, which is severely bradycardic. The ventricular rhythm originates from a ventricular pacemaker based on the wide QRS complexes. The key feature is AV dissociation, where the atrial and ventricular rhythms are independent, and the PR intervals vary without a predictable pattern.

A 62-year-old male with a history of hypertension and substance use disorder activated emergency medical services for two days of worsening chest pain with radiation to his back, accompanied by lightheadedness and a fall with head-strike. On emergency department (ED) arrival, the patient was found to be in obvious distress, endorsing ongoing chest pain and lightheadedness. On physical exam, he was diaphoretic and bradycardic with equal, thready pulses in all extremities. Initial vital signs showed a heart rate of 22 bpm and an undetectable blood pressure. An ECG was immediately obtained in the ED, revealing complete dissociation between atrial and ventricular activity consistent with CHB (Figure 1).

**Figure 2:**
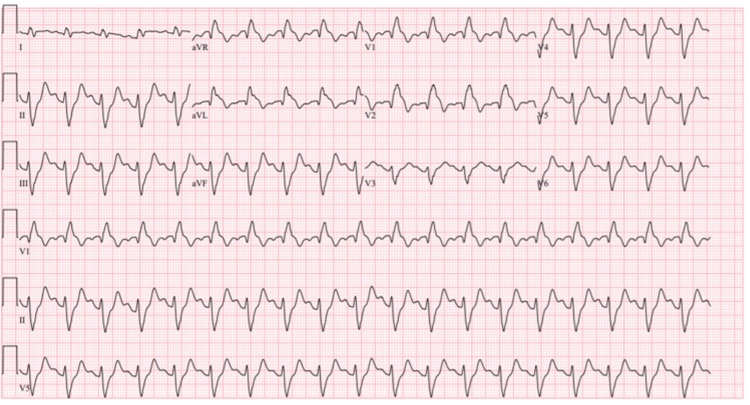
ECG obtained after conversion from ventricular tachycardia. This ECG shows sinus tachycardia with a right bundle branch block and left anterior fascicular block with ST-segment elevation in aVL and septal leads.

Given the patient's presentation and initial diagnostic findings, advanced cardiac life support (ACLS) was immediately initiated. Atropine was trialed with no effect, and transcutaneous pacing (TCP) was successfully initiated. The patient was started on an epinephrine infusion, and the catheterization laboratory was activated for coronary revascularization and emergent pacemaker placement. The patient was intubated for airway protection and facilitation of TCP, which improved capture. Unfortunately, the patient developed ventricular tachycardia, which spontaneously converted to sinus tachycardia with a right bundle branch block and left anterior fascicular block with ST-segment elevation in aVL and septal leads (Figure 2).

Due to concerns for ischemia, the patient underwent coronary arteriography, which revealed a complete proximal LAD artery occlusion. The interventionalist promptly placed a drug-eluting stent in the LAD to manage the occlusion, and a transvenous balloon pacemaker was inserted as bridge therapy to a definitive pacemaker for the ongoing dysrhythmias. Finally, an intra-aortic balloon pump was placed for management of cardiogenic shock.

## Discussion

CHB in the setting of an AMI, particularly due to LAD artery occlusion, is a rare but critical complication associated with high morbidity and mortality. CHB more commonly occurs with inferior wall myocardial infarctions due to right coronary artery (RCA) involvement. However, when associated with anterior wall infarctions, it often indicates extensive myocardial damage and a poorer prognosis [1]. The sinoatrial (SA) node is supplied by the RCA in 63-73% of cases and the left circumflex artery (LCX) in 27-37%, the AV node is supplied by the RCA in 80-90% of cases, while LCX supplies 10-20%, and lastly the bundle of his & bundle branches are supplied by the LAD [2].

Several studies have highlighted the increased risk of conduction disturbances in patients with AWMI. According to a recent analysis, CHB in the context of AWMI has a higher short-term mortality rate (as high as 60%) compared to inferior wall infarctions with CHB [3]. In general, patients who develop CHB after an AMI tend to have higher rates of left ventricular dysfunction and cardiogenic shock [4]. This is due to the larger infarct size, reduced left ventricular ejection fraction, and the increased likelihood of hemodynamic instability [4]. Additionally, a study analyzing the incidence of CHB in STEMI patients found that those with anterior infarctions were more likely to require temporary or permanent pacemaker implantation due to the persistence of conduction abnormalities [1].

The 2013 American College of Cardiology Foundation (ACCF)/American Heart Association (AHA) ST-elevation myocardial infarction (STEMI) guidelines recommend early revascularization in such cases to improve survival and reduce complications [5]. The management of CHB in AMI is multifaceted and depends on the hemodynamic stability of the patient. In this case, the patient’s presentation with profound bradycardia and hypotension necessitated immediate intervention with TCP followed by transvenous pacing. Atropine is generally ineffective in CHB secondary to extensive ischemia and was unsuccessful in this case. Emergent revascularization remains the cornerstone of management. PCI of the LAD has been shown to restore conduction in some cases, potentially obviating the need for permanent pacing [6].

## Conclusions

CHB in the setting of AWMI is rare but carries significant mortality due to rapid hemodynamic deterioration and extensive myocardial injury. Prompt recognition of conduction disturbances, especially in patients presenting with profound bradycardia and shock, is essential. Because ischemia-related CHB is often unresponsive to atropine, early pacing, initially transcutaneous, followed by transvenous if required, is critical for stabilization. Emergent coronary revascularization remains the cornerstone of therapy and may restore conduction while preventing further myocardial damage, potentially avoiding the need for permanent pacing. Heightened clinical vigilance and aggressive intervention are paramount to optimizing outcomes in this high-risk patient population.
